# DNA hydrolysing IgG catalytic antibodies: an emerging link between psychoses and autoimmunity

**DOI:** 10.1038/s41537-021-00143-6

**Published:** 2021-02-26

**Authors:** Rajendran Ramesh, Aparna Sundaresh, Ravi Philip Rajkumar, Vir Singh Negi, M. A. Vijayalakshmi, Rajagopal Krishnamoorthy, Ryad Tamouza, Marion Leboyer, A. S. Kamalanathan

**Affiliations:** 1 Centre for BioSeparation Technology, Vellore Institute of Technology (VIT), Vellore, Tamil Nadu India; 2 Department of Clinical Immunology, Jawaharlal Institute of Postgraduate Medical Education & Research (JIPMER), Puducherry, India; 3 Department of Psychiatry, Jawaharlal Institute of Postgraduate Medical Education & Research (JIPMER), Puducherry, India; 4 Fondation FondaMental, Créteil, France; 5 Department of Psychiatry and Addictology, Mondor University Hospital, AP-HP, DMU IMPACT, Créteil, France; 6 University Paris-Est-Créteil, UPEC, Creteil, France; 7 INSERM, U955, Mondor Institute for Biomedical Research, IMRB, Translational Psychiatry, Créteil, France

**Keywords:** Psychiatric disorders, Psychology

## Abstract

It is not uncommon to observe autoimmune comorbidities in a significant subset of patients with psychotic disorders, namely schizophrenia (SCZ) and bipolar disorder (BPD). To understand the autoimmune basis, the DNA abyzme activity mediated by serum polyclonal IgG Abs were examined in psychoses patients, quantitatively, by an in-house optimized DNase assay. A similar activity exhibited by IgG Abs from neuropsychiatric-systemic lupus erythematosus (NP-SLE) patients was used as a comparator. Our data revealed that the IgG DNase activity of SCZ was close to that of NP-SLE and it was twofold higher than the healthy controls. Interestingly, the association between DNase activity with PANSS (positive, general and total scores) and MADRS were noted in a subgroup of SCZ and BPD patients, respectively. In our study group, the levels of IL-6 and total IgG in BPD patients were higher than SCZ and healthy controls, indicating a relatively inflammatory nature in BPD, while autoimmune comorbidity was mainly observed in SCZ patients.

## Introduction

Growing evidence indicate that neuroinflammation and immune dysfunction play a role in the clinical manifestations of psychotic disorders *viz*. schizophrenia (SCZ) and bipolar disorder (BPD)^1–3^. Besides, frequent association of autoimmune comorbidities are also reported in psychotic disorders^4^. Raised levels of C-reactive protein and pro-inflammatory cytokines *viz*. interleukin-2 (IL-2), IL-6, interferon-γ, tumour necrosis factor-α, in the patients’ serum are markedly associated with psychotic syndromes i.e., mania, depression, etc^5^. Epidemiological studies emphasise the involvement of an autoimmune drive in the disease manifestations of SCZ and BPD patients^6^. From a clinical perspective, the psychotic disorders presents several parallels to autoimmune diseases like early-onset, immune dysregulation, the incidence of alternating active-remission disease courses and prevalence of autoantibodies (Aabs)^7^. Further, genetic association studies along with the impact of environmental factors (infectious stigma, social stress) raise the plausibility that the autoimmune response are either predisposed or proactive in these disorders^8,9^. Interestingly, administration of few immunosuppressive drugs, used in autoimmune pathologies, seems to attenuate psychotic symptoms in a subset of psychiatric patients^10^. The occurrence of Aabs in the blood of a significant subset of psychotic patients suggests the involvement of humoral autoimmunity, which might contribute to psychotic manifestations and disease severity^11,12^. Intriguingly, the occurrence of IgG Aabs against neurotransmitter receptors and other macromolecules (DNA, brain lipids, gangliosides, and cardiolipin) has been described in a proportion of SCZ and BPD patients^13,14^. Particularly, IgG Aabs against neuronal receptors like N-methyl-d-aspartate receptor (NMDAR), γ-aminobutyric acid receptor (GABAR), and α-amino-3-hydroxy-5-methyl-4-isoxazolepropionic acid receptor (AMPAR) have been observed during the psychotic crisis^15,16^. Also, a systematic study by Pearlman and Najjar revealed a high prevalence of anti-NMDAR IgG Abs in SCZ patients and to a lesser extent in BPD patients^17^.

Above facts suggest a potential overlap between psychotic syndromes and autoimmunity. Despite these clues, the knowledge about the autoimmune processes at play in psychiatric disorders remains elusive. Use of conventional diagnostic criteria, Diagnostic and Statistical Manual of Mental disorders (DSM) for the assessment of psychotic syndrome; presence or absence of Aabs, age of onset, clinical presentations and non-interventional clinical investigations, do not fully take into account the autoimmunity based differentiation of the syndromes. Such a gap led to the recent update of the criteria for the diagnosis and disease classification, published in the recent position paper by Pollack et al.^18^. This update takes into consideration the autoimmunity based risks in psychiatric syndrome as well as its influence on disease mediation. This has led to the establishment of a new disease category represented as “autoimmune psychosis”. Any biomarker that could stratify this category more objectively is of great interest not only to expand the horizon of autoimmune concept in a subset of psychoses, but also to tailor some evidence-based individualised therapeutic strategies.

Robust association of psychiatric symptoms in diverse autoimmune diseases *viz*. multiple sclerosis (MS), scleroderma, anti-phospholipid syndrome, and systemic lupus erythematosus (SLE) have also been reported^19,20^. Especially in case of MS and neuropsychiatric systemic lupus erythematosus (NP-SLE), along with conventional Aabs (anti-nuclear Abs, anti-dsDNA Abs), presence of neuronal Aabs (anti-NMDAR) is demonstrated and this may contribute to the observed psychotic/central nervous system (CNS) manifestations^21–24^.

The biological function of Aabs in autoimmune diseases, beyond simple antigen recognition, sometimes attributes for high order function i.e., catalysis. Fascinatingly, such cognate Abs exhibit proteolytic, DNase and RNase activity, which are well-proven to be an intrinsic property and such Abs are referred to as catalytic Abs or natural abzymes^25^. These Abs are capable of modifying the self-antigens (proteins and nucleic acids) chemically like that of naturally occurring enzymes^26^. Exposing the conserved or modified motifs of the endogenous antigens to the immune system can randomly elicit the generation of anti-idiotypic Abs (Aabs) endowed with enzymatic properties. It is very likely by this mechanism, the abzymes may participate not only in the host defence but also when go awry, can drive the inflammatory or autoimmune process. In autoimmune diseases like MS, thyroiditis, acquired haemophilia, SLE, rheumatoid arthritis, systemic sclerosis, the Abs not only recognise the endogenous macromolecules like myelin basic protein, thyroglobulin, Factor VIII, DNA and RNA molecules but also are capable of hydrolysing them^27–29^. Whether the evaluation of abzyme function would add value, directly or indirectly, to the current arsenal of psychosis criteria and/or for stratification of the psychosis spectrum, remains to be studied.

At this end, we initiated a comparative study to evaluate quantitatively the DNA abzyme function of IgG Abs from SCZ, BPD, and NP-SLE patients along with healthy controls. Additionally, anti-dsDNA Abs titre, total IgG, and IL-6 levels were measured and compared in three disease groups along with healthy controls. Further, the relationship between DNase activity with psychotic disease scores and immunological parameters were examined, to decipher the association of autoimmune spectrum in psychoses.

## Results

### Clinical profile

Table 1 briefs the biochemical and demographic profile of the study participants. Table 2 represents the inflammation status (IL-6, serum IgG level) and autoantibody profile (ANA, anti-dsDNA) of the patients.

**Table 1:** Demographic and clinical profile.

Patients and variables	SCZ (*n* = 31)	BPD (*n* = 31)	NP-SLE (*n* = 20)	HC (*n* = 25)
*Demographic*				
Age	35.1 ± 7.5^a^	31.2 ± 9.9	28 ± 9.13	34.9 ± 8.1
Sex (% F)	58	52	95	52
Body mass index	23.15 ± 4.9	22.3 ± 3.8	–	23.1 ± 3.6
Disease status—acute (%)	58	61	Active CNS disease	–
Disease duration (months)	10.1 ± 9.9	5.5 ± 6.7	13.6 ± 6.5	–
Smoking (%)	25.8 (8)	22.6 (7)	0	12
Age of onset (years)	26.88 ± 7.8	22.4 ± 4.7	27.6 ± 2.7	–
*Medication*^b^				
Antipsychotic typical (%)	54.83	68	–	–
Antipsychotic atypical (%)	45.16	20	–	–
Mood stabiliser (%)	3.2	92	–	–
Immunomodulators/Immunosupressive drugs (%)	–	–	65	–
Mycofenolate Mofetil (%)	–	–	15	–
Cyclophosphamide (%)	–	–	20	–
Methotrexate (%)	–	–	20	–
Steroids (%)	–	–	50	–
*Measure of symptoms*				
PANSS total	64.4 ± 23.2	–	–	–
MADRS	–	3.4 ± 6.5	–	–
YMRS	–	15.5 ± 10.5	–	–
FAST	42.06 ± 22.8	42.6 ± 15.1	–	–
GAF	45.4 ± 20.7	49.7 ± 12.3	–	–
*Infectious agents*				
HERV-W % positive (*n*)	6.5 (2)	3.2 (1)	–	–
CMV % positive (*n*)	16 (5)	25.8 (8)	–	4 (1)
Toxoplasma Gondi % positive (*n*)	22.6 (7)	12.9 (4)	–	8 (2)

*SCZ* schizophrenia, *BPD* bipolar disorder, *HC* healthy controls, *NP-SLE* neuropsychiatric-systemic lupus erythematosus, *PANSS*, positive and negative syndrome scale, *MADRS* Montgomery and Asberg depression rating scale, *YMRS* Young Mania rating scale, *FAST* functioning assessment short test, *GAF* global assessment of functioning, *HERV-W* human endogenous retrovirus-W, *CMV* cytomegalovirus.

^a^All the values are expressed as mean ± sd.

^b^Information for six BPD samples are missing.

**Table 2:** Immunological analysis.

Patients and variables	SCZ, (*n* = 31)	BPD, (*n* = 31)	HC, (*n* = 25)	NP-SLE, (*n* = 20)	Statistical significance^1^
*Inflammation parameters*					
IL-6 (pg/mL)	9.3 ± 0.87	12.35 ± 1.04	6.45 ± 1.16	–	BPD vs. HC***^b^ ^b^SCZ vs. HC**^b^
Total IgG (mg/mL)	11.59 ± 0.76	12.87 ± 0.78	9.96 ± 0.49	12.39 ± 1.17	^a^BPD vs. HC**
*Autoantibodies*					
Anti-nuclear antibody % positive (*n*)	12.9 (4)	9.7 (3)	0 (0)	100 (20)	–
Anti-neuronal antibody % positive (*n*)	6.5 (2)	3.2 (1)	4 (1)	–	–
Anti-dsDNA (IU/mL)	10.57 ± 1.46	9.94 ± 1.26	12.4 ± 1.07	72.93 ± 17.13	SCZ vs. NP-SLE****^b^ BPD vs. NP-SLE****^b^

****p < 0.0001, ***p = 0.0001, **p < 0.01.

^1^Values are expressed as mean ± SEM.

^a^Student’s *t*-test.

^b^Mann–Whitney *U*-test.

### Purification of IgG Abs

**Fig. 1:**
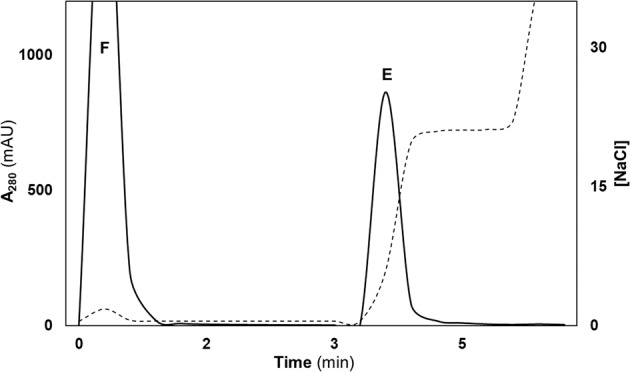
A typical chromatogram of total IgG purified from serum on a CIM-histidyl disk. About 150 µL serum (tenfold dilution) in binding buffer, 25 mM MOPS buffer pH 6.5 was injected and the bound proteins were eluted with 25 mM MOPS pH 6.5 + 0.4 M NaCl. Flow rate: 4 mL/min. Peaks, F and E represent flow-through and elution, respectively.

**Fig. 2:**
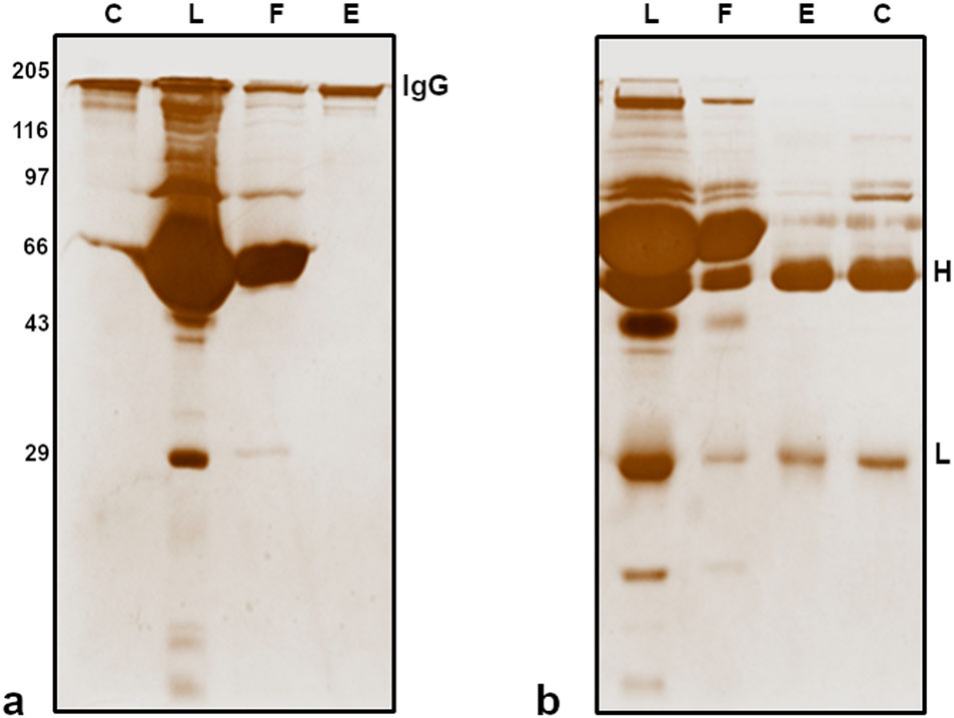
SDS-PAGE (10%) analysis. **a** Non-reducing conditions, **b** reducing conditions. Lanes: C-human IgG, Cohn fraction (II, III), L-total serum, F-flow-through and E-elution (0.2 M NaCl). H and L correspond to heavy chain (50 KDa) and light chain (25 KDa) of IgG. Gels were derived from the same experiment.

To investigate the presence of DNA hydrolysing activity, polyclonal IgG Abs were recovered from the serum of SCZ, BPD, NP-SLE patients, and the healthy controls. Here, CIM^®^ disk coupled with the bio-affinity ligand, l-histidine, was used for the recovery of total IgG Abs directly from the serum, in a single-step (Fig. 1). The SDS-PAGE analysis (Fig. 2a) under non-reducing conditions revealed an intact IgG molecule (150 KDa) in the eluted peak fractions (Fig. 1, label E). As expected, under reducing conditions (Fig. 2b) two bands corresponding to heavy (50 KDa) and light chains (25 KDa) of IgG Abs were prominent. Under similar conditions the therapeutic intravenous immunoglobulin (IVIg) was also subjected to purification. Overall, the antibody preparation was found to be homogenous and was used for further analysis.

### DNA hydrolysis assay

**Fig. 3:**
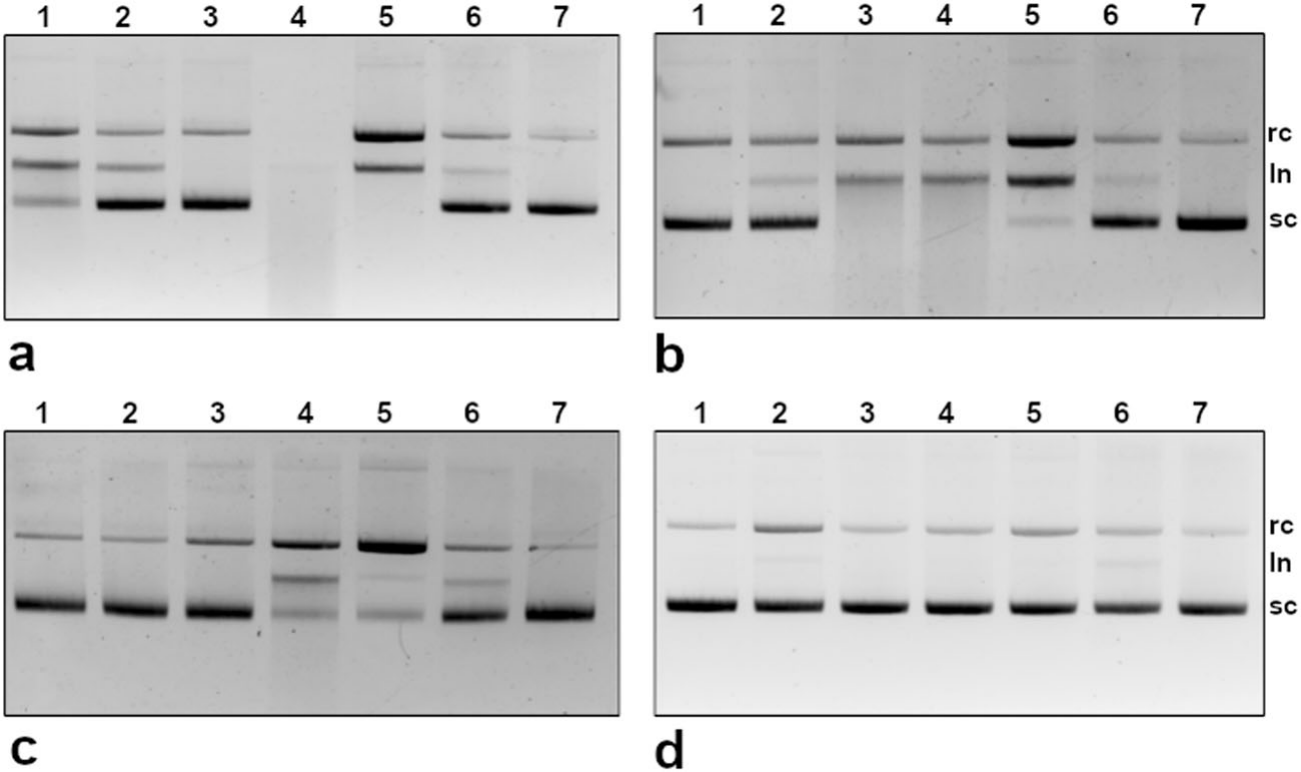
DNA hydrolysis by total IgG antibody. **a**–**d** Neuropsychiatric systemic lupus erythematosus (NP-SLE), schizophrenia (SCZ), bipolar disorder (BPD), and healthy control (HC) samples, respectively. Plasmid DNA, pUC18 (50 ng) was incubated with IgG (2 µg) for 2 h at 37 °C and visualised by ethidium bromide stain. Lanes: 1–5, IgG from patients; 6, IVIg & 7, DNA incubated alone. sc supercoiled DNA, rc relaxed circular DNA, ln linear DNA.

The DNA hydrolysing activity by polyclonal IgG Abs from the SCZ, BPD, and NP-SLE patients along with the healthy controls are illustrated in Fig. 3. Remarkably, both SCZ and BPD IgG Abs revealed DNA hydrolysing activity while the healthy controls and IVIg showed a non-negligible activity. The plasmid DNA hydrolysis mediated by IgG Abs progressed in a pronounced manner with relative disappearance of the supercoiled form (sc) and the appearance of the relaxed form (rc) followed by linear form (ln), notably in the SCZ, BPD, and NP-SLE samples. However, the IgG Abs from the disease groups exhibited differences in the rate of hydrolysis, wherein few SCZ and NP-SLE samples showed rapid hydrolysis of the DNA.

### Antibody concentration and time-dependent assay for DNA hydrolysis

**Fig. 4:**
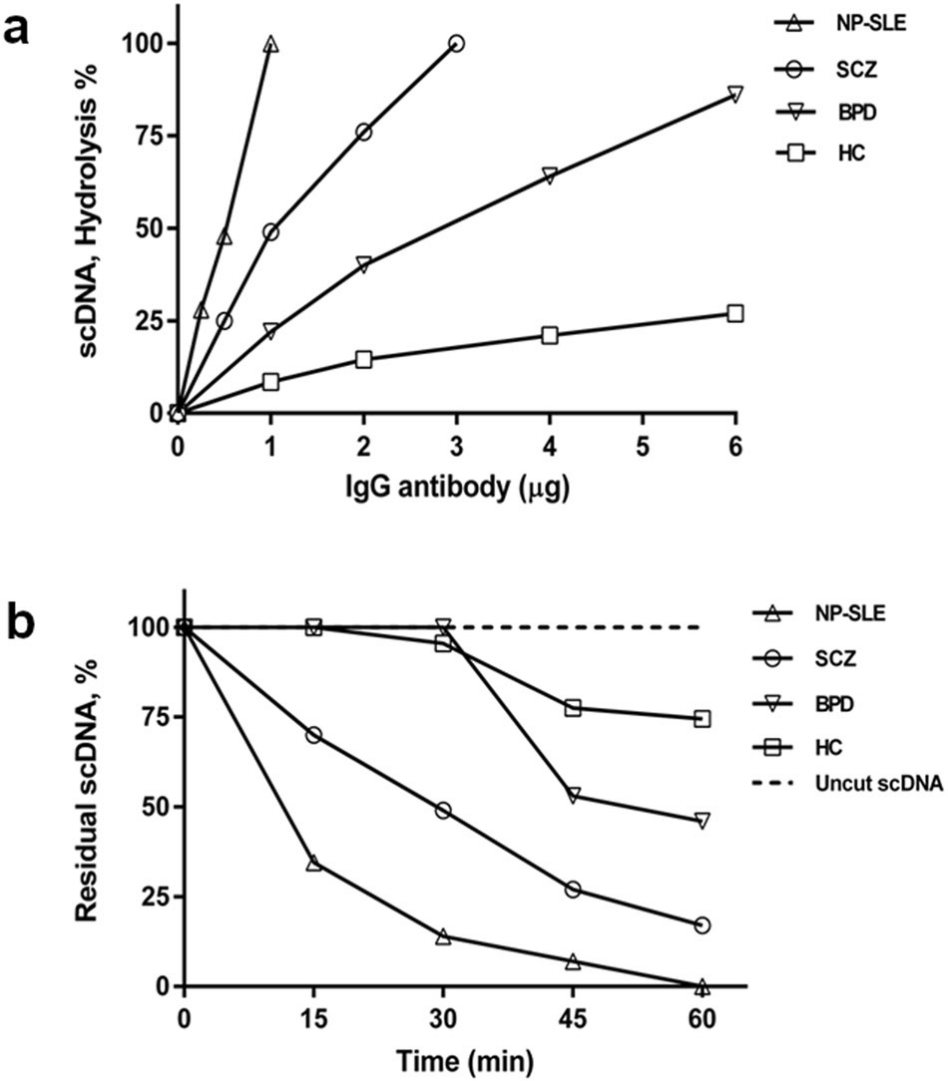
Antibody concentration and time-dependent optimisation assays. **a** Optimisation of IgG concentration: plasmid DNA (50 ng) was incubated with increasing concentration of IgG (1–6 µg) for 2 h at 37 °C. **b** Time-point assay: fixed concentration of IgG (1 µg) was incubated with plasmid DNA (50 ng). Aliquots were analysed on an agarose gel (0.8%) and the scDNA hydrolysis was estimated by densitometry analysis (Image Lab^TM^ 6.0.1, BioRad). NP-SLE neuropsychiatric systemic lupus erythematosus, SCZ schizophrenia, BPD bipolar disorder, HC healthy control.

To measure IgG mediated DNA hydrolysis quantitatively, the IgG Abs concentration and time course required for the hydrolysis of a fixed concentration of DNA were determined. First, we determined the IgG Abs concentration required for hydrolysing DNA in each study group. Figure 4a is a schematic representation of the DNA hydrolysed by increasing concentration of affinity purified IgG Abs from the disease groups and the healthy controls. Agarose gel data (not shown) was used to compute the hydrolysis percentage, mainly the scDNA, by densitometry analysis. Results revealed that 1 µg of IgG Abs were adequate to quantitatively measure the scDNA hydrolysis.

Subsequently, time point assay (Fig. 4b) was performed with 1 µg IgG Abs from disease samples and healthy controls. In the agarose gel (not shown), a pattern of steady decrease (since hydrolysed) in the scDNA was noted with increasing time of incubation that was quantified by densitometry. The percentage hydrolysis was calculated by subtracting the quantified values of residual scDNA from the amount of intact scDNA (incubated without IgG Abs), at respective time points. Interestingly, the activity data of IgG Abs from the SCZ group was proportionately close to the activity exhibited by IgG Abs from the NP-SLE group.

**Fig. 5:**
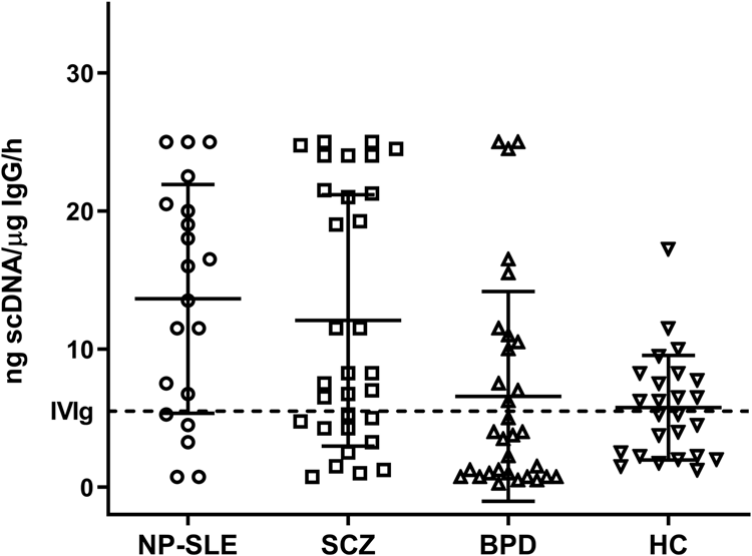
Comparative analysis of IgG mediated DNA hydrolysis. IgG (1 µg) was incubated with plasmid DNA (50 ng) for 1 h at 37 °C and hydrolysed products were analysed on 0.8% agarose gel. scDNA hydrolysis was determined and calculated by densitometry analysis (Image Lab^TM^ 6.0.1, Bio-Rad). One-way ANOVA analysis revealed a significance between the groups with *F* (3, 103) = 6.792, *p* = 0.0003. IVIg (-----), NP-SLE neuropsychiatric systemic lupus erythematosus (*n* = 20), SCZ schizophrenia (*n* = 31), BPD bipolar disorder (*n* = 31), HC healthy control (*n* = 25).

These functional assays allowed to quantitate the IgG mediated DNA hydrolysing activity (ng DNA/µg IgG Abs/1 h) exhibited by patients in the disease groups, healthy controls and IVIg sample (Fig. 5). One-way ANOVA analysis was used for the comparison of the activity between the four groups. The ANOVA analysis revealed significance with *p* = 0.0003 and degree of freedom *F* (3,103) = 6.792. Based on the central tendency value (mean ± sd) highest DNase activity, as expected, was noted in NP-SLE (13.6 ± 8.7) and successively followed by SCZ (12.1 ± 9.2), BPD (6.5 ± 7.6) and healthy control (5.7 ± 3.7).

Of further interest, the immunological analysis revealed a low titre of anti-dsDNA Abs in both SCZ and BPD patients on contrary to NP-SLE patients (Table 2). Both the IL-6 and IgG levels exemplify an underlying inflammatory condition in our BPD participants compared to SCZ and health controls (Table 2). This data suggests presence of neuro-immuno-inflammation that is concomitant with the previously reported observations^5,30,31^.

**Fig. 6:**
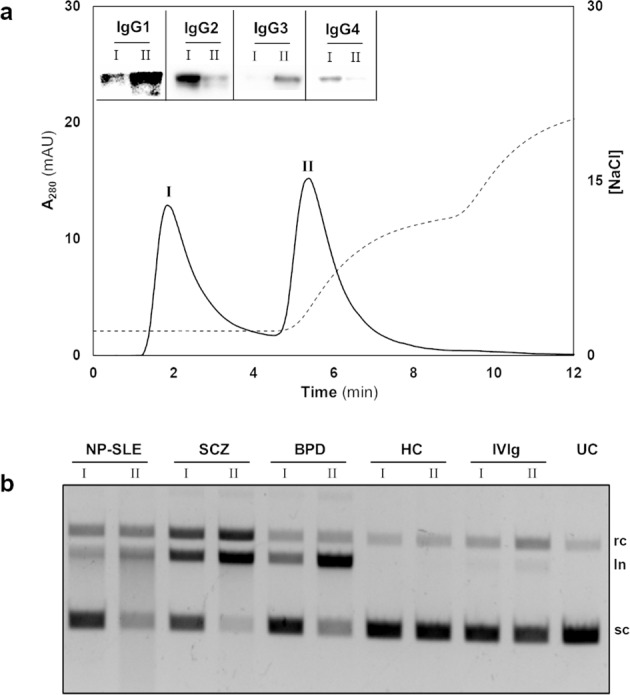
IgG subclasses separation and DNA hydrolysis. **a** About 50 µg of pre-purified total IgG Abs (twofold dilution) suspended in 25 mM Tris-HCl pH 7.4 was fractionated and eluted using 25 mM Tris-HCl pH 7.4 + 0.2 M NaCl. Peak I-flow-through, Peak II-elution. Inset: IgG subclasses in the peak I and II were discriminated by separate western blots. **b** DNA hydrolysis exhibited by peaks I (lane I) and II (lane II). UC- scDNA incubated alone; sc supercoiled DNA, rc relaxed circular DNA, ln linear DNA.

The efficiency of l-histidine ligand to discriminate the IgG subclasses as described elsewhere^32^ was employed, to evaluate the DNA hydrolysing activity by the IgG subclasses. Figure 6a represents the chromatogram of IgG subclasses. Western blot analysis (Fig. 6a, inset) confirmed the presence of IgG2 subclass in peak I (flow-through) and IgG1 subclass in peak II (elution) fractions, respectively. Interestingly, the peak II fractions, comprising IgG1 subclass, attributed for the DNA hydrolysis in the NP-SLE, SCZ and BPD samples (Fig. 6b). On the contrary, the peak I fraction (IgG2 subclass) revealed no activity in the disease groups. However, no activity was observed in healthy controls and IVIg.

## Discussion

To further refine the autoimmune features in SCZ and BPD, herein, we investigated the IgG DNA abzyme activity in these patients and compared the activity profile with the IgG from NP-SLE patients. We chose to employ judiciously NP-SLE as a positive control, due to following reasons (i) evidence of association of psychiatric symptoms in a subset of SLE patients^20^, (ii) prevalence of anti-dsDNA Abs in the circulation^33^, and (iii) presence of DNase activity in their IgG Abs^27^. The bio-affinity l-histidyl chromatographic system was used due to its efficiency in recovering structurally intact and electrophoretically homogenous IgG Abs and its subclasses^32,34^. To assess the IgG mediated DNase activity quantitatively, our experimental conditions were optimised to determine the antibody concentration and time course required for the scDNA hydrolysis (Fig. 4). Interestingly, the close resembles of DNase activity between SCZ and NP-SLE (Fig. 5) highlights partially that the IgG Abs from SCZ shares autoimmune-like features. Besides, the SCZ IgG Abs showed a twofold higher activity than the healthy controls. Our findings corroborate with a recent report by Ermakov et al.^35^ describing the presence of IgG DNA abzyme, studied exclusively in SCZ patients. Nevertheless, our comparative analysis of DNA hydrolysing IgG Abs (Fig. 5) from the SCZ, BPD, NP-SLE patients, and healthy controls bring robust evidence of autoimmune association not only in SCZ but also broadly in psychoses per se. Nevertheless, it was surprising to note a twofold lower DNase activity in our BPD patients compared to NP-SLE. Unexpectedly, a subset of BPD patients (41.9%) showed DNase activity less than that of the healthy controls and IVIg. Observations of the low activity may be due to heterogeneity among the BPD patients, which hypothetically could stem from either disease severity or status of mood oscillation or effect and duration of medications that requires further insight.

Despite presence of a statistical significance among the four groups (one-way ANOVA analysis), the distribution pattern of DNase activity was surprisingly uneven in SCZ and BPD groups (Fig. 5). Based on the distribution, the above patient groups were further divided into two subgroups, namely “above-average subgroup” and “below-average subgroup” by fixing the mean as a cut-off. Subsequently, the two subgroups were analysed for a potential relationship between the psychotic disease scores and immunological parameters (Table 3). Interestingly, the SCZ above-average subgroup showed a positive association with the PANSS-positive, general and total scores, while MADRS associated with the BPD above-average subgroup. Consequently, other variables between the two subgroups in the SCZ and BPD showed moderate differences without statistical significance. These data are likely to open windows for eventual patient stratification provided a much larger study is carried out with a range of psychosis spectrums, to draw appropriate conclusions.

**Table 3:** Subgroup division based on DNA hydrolysis distribution.

	Schizophrenia		Bipolar disorder	
Scores and variables	Above-average subgroup (*n* = 12)	Below-average subgroup (*n* = 19)	Above-average subgroup (*n* = 11)	Below-average subgroup (*n* = 20)
PANSS positive	16.1 ± 5.8^a^	12.95 ± 6.2	–	–
PANSS negative	17.2 ± 8.4	18.35 ± 9	–	–
PANSS general	35.4 ± 9	30.5 ± 10.9	–	–
PANSS total	68.7 ± 19.3	61.3 ± 23.8	–	–
CDSS^b^	1 ± 1.6	1.3 ± 2.0	–	–
MADRS^c^	–	–	6.7 ± 10	2.33 ± 4.3
YMRS^d^	–	–	13.28 ± 13	14.87 ± 8.8
GAF	39.4 ± 18.4	48 ± 20.7	50.85 ± 11.1	51.27 ± 12.6
IL-6	5.5 ± 2.9	6 ± 5.6	7.4 ± 3.5	6.6 ± 5.8
Total IgG	11.2 ± 4.99	11.5 ± 4.16	13.25 ± 4.5	12.6 ± 4.2
Disease duration (months)	8.8 ± 7.5	8.35 ± 7.2	6.4 ± 8.5	5.07 ± 6

^a^All values are expressed as mean ± sd.

^b^Calgary depression scale for schizophrenia.

^c,d^Information for six BPD samples are missing.

There are some limitations to this study. First, the sample size is small and requires to be assessed with a much larger clinically well-defined cohort. Eventually, this will help in exploring a plausible spatiotemporal association between the disease characteristics and the IgG mediated activity. Second, the influence of medications on the activity along with the disease features is not explored. Third, concurrent analyses of CSF and serum samples may be warranted to examine the precise link between autoimmune process and episodes of psychosis.

Despite small sample size, our preliminary case-control results highlight the presence of DNA abzyme activity, in general, in psychoses and prominently in SCZ patients. Overall, our findings support for prospective autoimmune spectrum in SCZ and BPD patients. Altogether capability to proactively identify and stratify the psychotic patients, assisted by clinical, biological and genetic features, may contribute to improve patient care.

## Methods

### Study design and participants

The objective of the present study was to evaluate the presence or absence of autoimmune comorbidity, based on catalytic antibody perspective, in SCZ and BPD patients. This was an experimental design study composed of case–control, recruited at Jawaharlal Institute of Postgraduate Medical Education & Research (JIPMER), India. The study protocol was approved by Institutional Ethics committee of JIPMER (ECR/342/Inst/PY/2013) and Vellore Institute of Technology (VIT) (IECH/2014/May23/05). Written informed consent was obtained from all the participants. The psychoses participants for the study were randomly selected from the individuals attending out-patient or in-patient services at the department of psychiatry. Patients diagnosed as SCZ and BPD, respectively and who fulfilled the study inclusion criteria i.e., Diagnostics and Statistical Manual of Mental disorder, IV edition (DSM IV)^36^, absence of any autoimmune disorder, inflammatory, and other neurological disorders were only recruited. A subset of systemic lupus patients with diffused psychiatric/neuropsychological syndromes^37^ (anxiety disorder, acute confusion status, cognitive disorder, mood disorder, and psychosis) were enrolled as disease comparator group at the department of clinical immunology. The clinical, demographic, biochemical, and medication profiles were collected at the time of enrolment from all the participants (Table 1), and were blinded to the investigators performing DNA abzyme studies until the analyses were completed. About 0.5 mL of serum was prepared from a non-fasting blood collected from the SCZ (*n* = 31), BPD (*n* = 31), and NP-SLE (*n* = 20) patients, respectively. Healthy controls (*n* = 25) without a personal or family history of psychotic disorders and autoimmune disorders were also recruited at JIPMER. All the samples were of south Indian ethnicity.

### IgG antibody purification

The bio-affinity ligand l-histidine was covalently coupled to CIM^®^ monolithic disk (BIA Separations d.o.o, Slovenia) bearing CDI chemistry^34^ to obtain a CIM-histidyl column. This chromatographic column was used for the purification of IgG Abs directly from serum. The purification experiment was carried out with an automated AKTA FPLC system (GE Healthcare, USA), integrated with a binary pump, UV detector, conductivity and fraction collector. The operation and acquisition of data were controlled by inbuilt UNICORN 5.0 software.

Briefly, about 150 µL of serum diluted tenfold in 25 mM MOPS buffer pH 6.5 was injected into CIM-histidyl column that was pre-equilibrated with the same buffer at a flow rate of 4 mL/min. The unbound proteins were washed away with the above buffer until the UV absorbance (280 nm) reached the baseline. The adsorbed proteins were eluted in a step gradient manner using NaCl (0.2 and 0.4 M) added in 25 mM MOPS buffer pH 6.5. Successively, the column was regenerated using 0.5 M NaOH. The eluted fractions were desalted and concentrated using Amicon Ultra-4 10 K centrifugal filter unit (Merck Millipore Bioscience, India). Protein concentration was determined by Bradford assay. The purity of IgG was analysed on SDS-PAGE (10%) under both non-reducing and reducing (DTT treatment) conditions. The gels were derived from the same experiments and they were processed in parallel. Under similar conditions IVIg (GAMUNEX-C, Girfols Inc., USA) was also subjected to purification and analysis.

### IgG subclass purification

Novarose^®^ (Inovata, Sweden) matrix immobilised with bio-affinity ligand l-histidine was used for the separation of IgG subclasses, as demonstrated earlier^32^. The pre-purified total IgG Abs (above section) was buffer exchanged with 25 mM Tris-HCl buffer pH 7.4, using Amicon Ultra-4 10 K device. The Novarose histidyl gel was packed into a glass column (1 × 1 cm) and was equilibrated with 25 mM Tris-HCl buffer pH 7.4. The enriched total IgG Abs (50 µg/50 μl) was diluted twofold in the equilibration buffer and re-chromatographed on Novarose histidyl column. The bound proteins were eluted using 0.1 and 0.2 M NaCl dissolved in the equilibration buffer in a step gradient manner. A flow rate of 1 mL/min was used and fractions of 0.25 mL were collected. Both, the flow-through and the eluted fractions were concentrated using Amicon Ultra-4 10 K device and subjected to SDS-PAGE analysis (10%) under non-reducing conditions. Individual, IgG subclasses were identified by western blot using human IgG subclass specific HRP conjugated Abs (Janssen Biochemica, Belgium) at a dilution of 1:10,000, which were processed with the study controls. Chemiluminescent substrate (BioRad, USA) was used for detection.

### IL-6 and anti-dsDNA measurement

The IL-6 was measured using Human IL-6 ELISA Kit (Diaclone, France) as per manufacturer instructions. IMTEC-dsDNA-Antibodies ELISA kit (Germany) was used for measuring anti-dsDNA titre, according to the manufacturer instructions.

### Serum total IgG measurement

Maxisorp 96-well plate (Nunc, Denmark) was coated with goat anti-human γ-chain specific Abs (P.A.R.I.S, France) diluted at 1:30,000 with phosphate-buffered saline, pH7.4 (PBS). Following overnight incubation at 4 °C, the plate was washed with PBS/0.05% V/V Tween20 (PBS-T) and blocked with 5% (w/v) skimmed milk for 2 h at 37 °C. About 100 µL of diluted (1:40,000 in PBS) patient and healthy serum were added to individual wells and the plate was incubated at 37 °C for 1 h. Next, the plate was washed and probed for 1 h with rabbit antihuman IgG HRP conjugate Abs (Sigma, USA) at 37 °C. The chromogenic substrate, tetramethylbenzidine (TMB) (Merck, India) was used for detection. The microplate was read at 450 nm using Enspire microplate reader (PerkinElmer, USA). Experiments were performed in duplicates and the absorbance was noted. Total human IgG Abs (Cohn fraction II, III) (Sigma, USA) was used to plot a standard curve. The concentration of total IgG in the patient groups, as well as healthy control serum was determined from the standard curve.

### DNA hydrolysis assay

The assay was performed in a 20 µL reaction mixture consisting of 50 ng of plasmid DNA pUC18 (>80% supercoil form) (Merck, India) and 2 µg of affinity purified total IgG Abs from the patient and control samples, respectively^27^. The reaction buffer comprised 20 mM Tris-HCl pH 7.5, 1 mM ethylenediaminetetraacetic acid (EDTA) and 5 mM MgCl_2_. As controls, IVIg with DNA and DNA alone in the reaction buffer were incubated along with other samples. The assay was performed at 37 °C for 2 h. The IgG subclass fractions obtained from re-chromatographed experiments were subjected to DNA hydrolysis assay under similar conditions except for the incubation time that was 1 h.

The digested DNA products were resolved on a 0.8% agarose gel electrophoresis (AGE) and were visualised by ethidium bromide stain. The gel image was captured using Chemidoc^TM^ MP imaging system.

### Concentration and time-dependent assays

For the concentration assay, about 50 ng of plasmid DNA pUC18 (substrate) was added to the increasing concentration of total IgG Abs (1–6 µg) in a 20 µL reaction buffer (20 mM Tris-HCl pH 7.5, 1 mM EDTA and 5 mM MgCl_2_). The reaction mixture was incubated for 2 h at 37 °C.

For the time-dependent assay, total IgG Abs (5 µg/100 µL) and plasmid DNA, pUC18 (250 ng/100 µL) were constituted in 100 µL reaction buffer and was incubated at 37 °C for 1 h. Aliquots of 20 µL were drawn at the time intervals of 0, 15, 30, 45, and 60 min and the reaction was stopped by deep freezing. These assays were performed with IgG Abs from the disease groups and healthy control.

Finally, the digested DNA products were resolved on 0.8% AGE, visualised by ethidium bromide stain and image was captured using gel-doc. The density of the intact scDNA was fixed as a standard (100%) to calculate the percentage hydrolysis of scDNA mediated by IgG Abs in a relative quantity mode using Image lab^TM^ software.

### Statistical analysis

Shapiro–Wilk’s test was used to check the Gaussian distribution of the analysed data. Samples that met the criteria of Gaussian distribution were analysed by Student’s *t*-test and those that did not meet the criteria were analysed by Mann–Whitney *U*-test. IgG mediated DNA hydrolysis of the four groups were analysed by one-way ANOVA test. GraphPad Prism 6 was used for statistically analysis and *p* < 0.05 was considered significant.

### Reporting summary

Further information on research design is available in the Nature Research Reporting Summary linked to this article.

## Supplementary information

Supplementary Information

REPORTING SUMMARY

## Data Availability

The data that supports the findings of this study are available from the corresponding author upon reasonable request and will be made available with prior permission from the JIPMER, India.
