# Supplementary material for: DNA hydrolysing IgG catalytic antibodies: an emerging link between psychoses and autoimmunity
Source: NPJ Schizophr. 2021 Feb 26;7:13. doi: 10.1038/s41537-021-00143-6 (PMC7910540; doi:10.1038/s41537-021-00143-6)
Supplement: Supplementary file 1 — Supplementary Information [file 41537_2021_143_MOESM1_ESM.pdf]

Supplementary Information

DNA hydrolysing IgG catalytic antibodies: An emerging link between psychoses and autoimmunity

Rajendran Ramesh[a], Aparna Sundaresh[b], Ravi Philip Rajkumar[c], Vir Singh Negi[b], Vijayalakshmi M.A[a], Rajagopal Krishnamoorthy[d], Ryad Tamouza [d,e,f,g], Marion Leboyer [d,e,f,g], Kamalanathan A.S[a] *

[a]Centre for BioSeparation Technology, Vellore Institute of Technology (VIT), Vellore, Tamil Nadu 632014, India

[b]Department of Clinical Immunology, Jawaharlal Institute of Postgraduate Medical Education & Research (JIPMER), Puducherry 605006, India

[c]Department of Psychiatry, Jawaharlal Institute of Postgraduate Medical Education & Research (JIPMER), Puducherry 605006, India

[d]Fondation FondaMental, Créteil, France

[e]Department of Psychiatry and Addictology, Mondor University Hospital, AP-HP, DMU IMPACT

[f]University Paris-Est-Créteil, UPEC, Creteil, France

[g]INSERM, U955, Mondor Institute for Biomedical Research, IMRB, Translational Psychiatry, Créteil, France

*** Corresponding author**

Dr. Kamalanathan A.S

Centre for BioSeparation Technology,

Vellore Institute of Technology (VIT), Vellore- 632014, Tamil Nadu, India

Email: kamalanathan_as@yahoo.com, kamal.as@vit.ac.in

Raw data of the gels and western blots

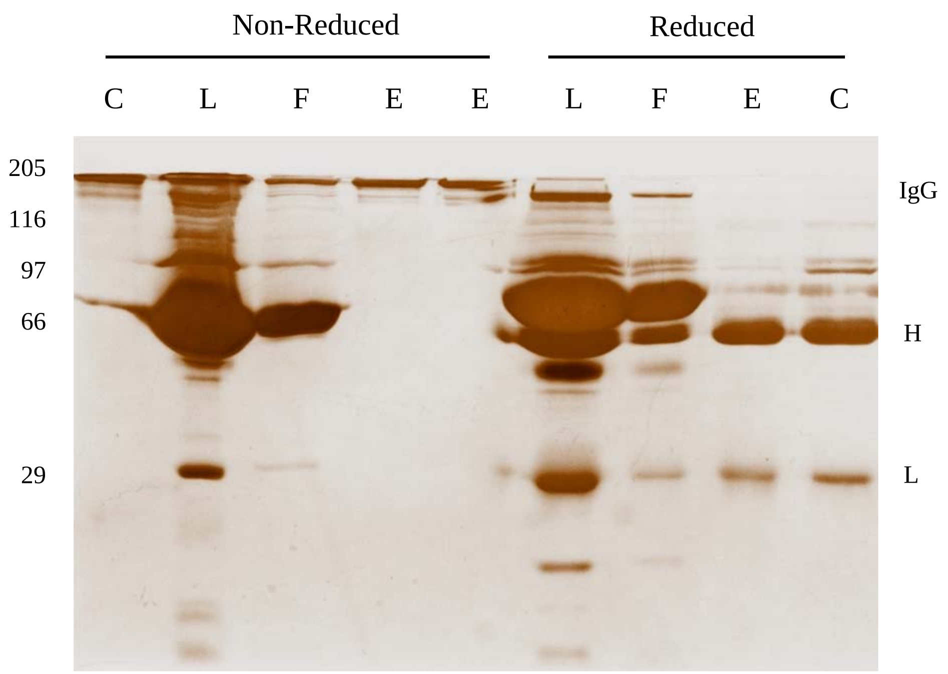

Figure2. **SDS-PAGE (10%) analysis.** Purified IgG were analysed under non-reducing conditions and reducing conditions. The gel is an unprocessed image. Lanes: C- human IgG, Cohn fraction (II, III), L- total serum, F- flow-through and E – elution (0.2M NaCl). H and L corresponds to heavy chain (50 KDa) and light chain (25 KDa) of IgG.

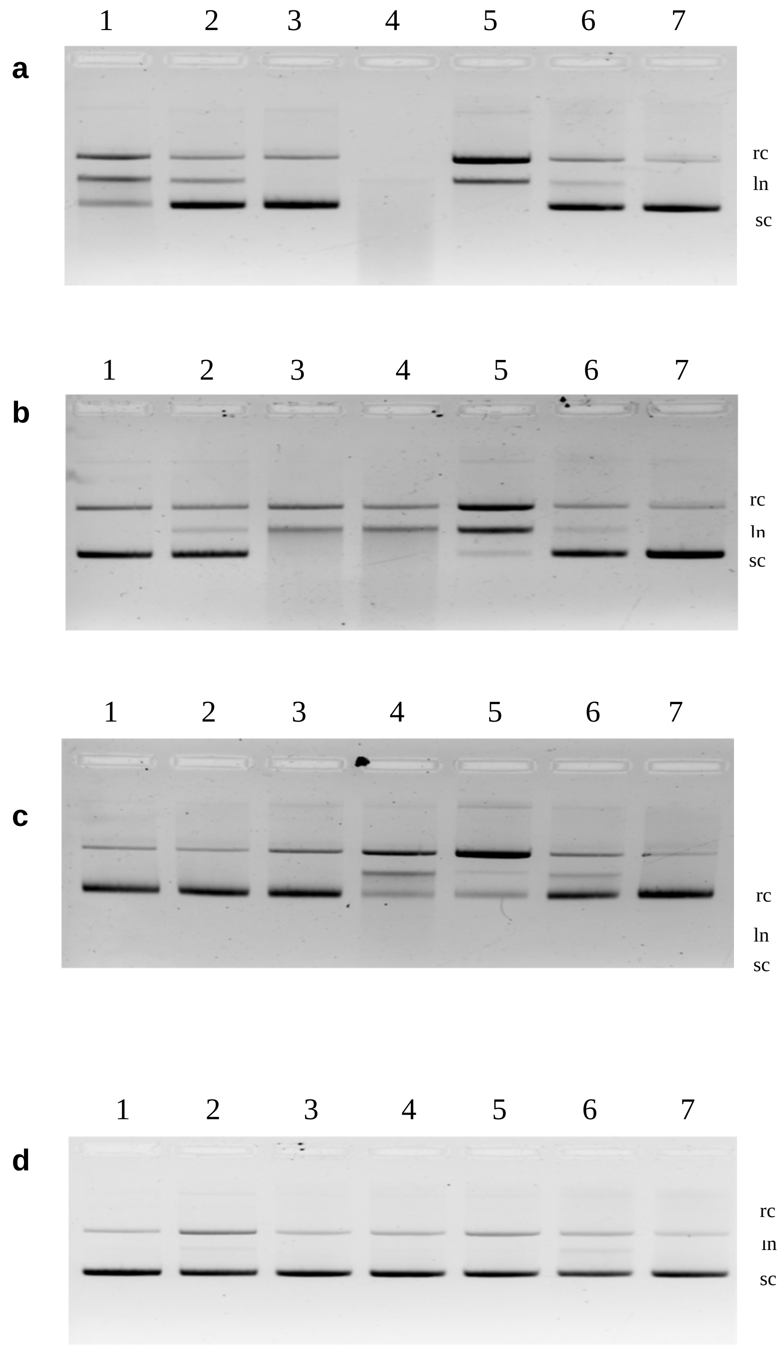

Figure3. **DNA hydrolysis by total IgG antibody**. **a, b, c** and **d** represent DNA hydrolysis by NP-SLE, SCZ, BPD and HC samples, respectively. Plasmid DNA, pUC18 (50ng) was incubated with IgG (2µg) for 2h at 37°C and visualised by ethidium bromide stain. Lanes: 1-5, IgG from patients; 6, IVIg & 7, DNA incubated alone. sc - Supercoiled DNA, rc - Relaxed circular DNA and ln - Linear DNA. NP-SLE, neuropsychiatric systemic lupus erythematosus, SCZ, schizophrenia, BPD, bipolar disorder, HC, healthy control.

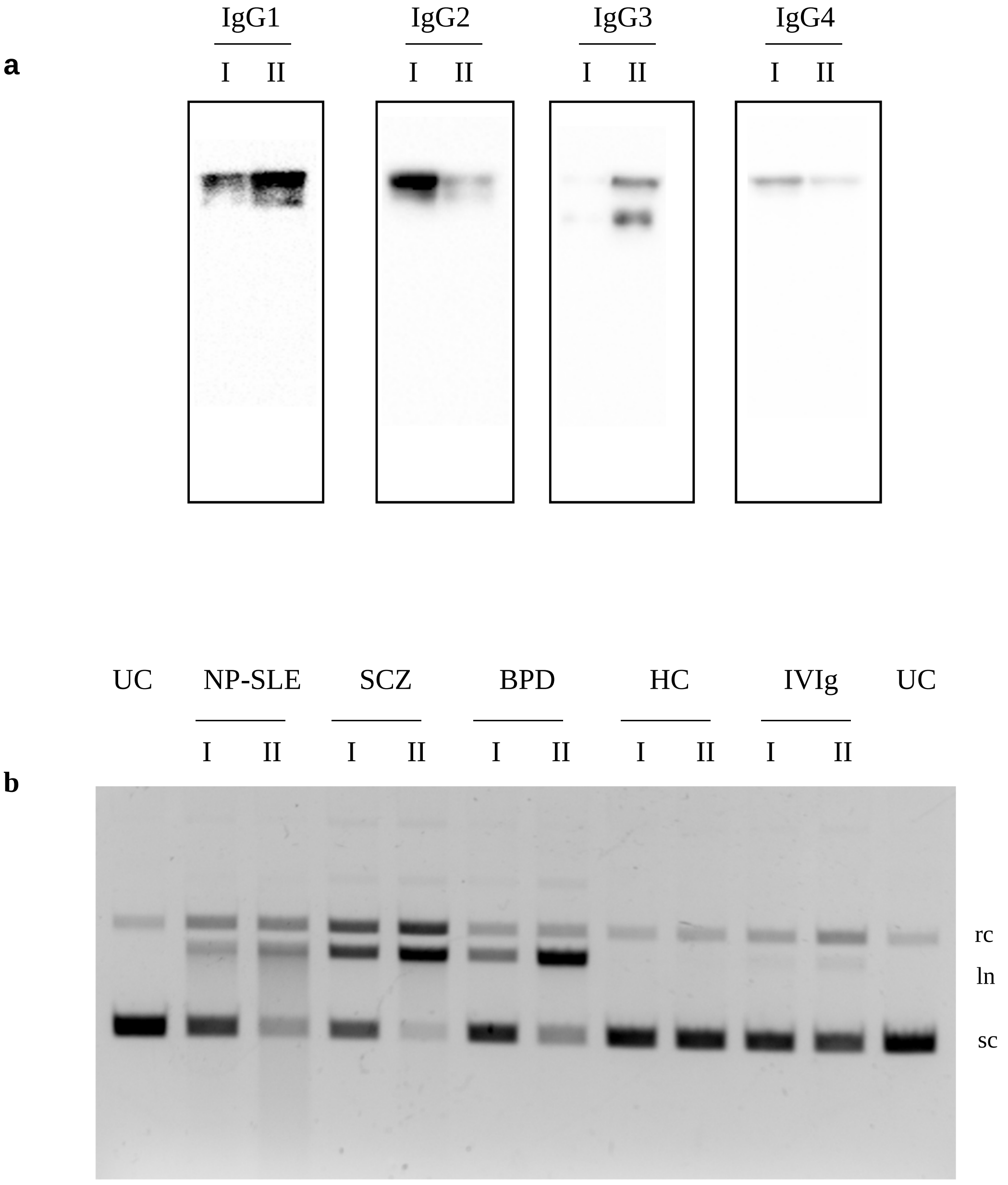

Figure6. Western blots of IgG subclasses and DNA hydrolysis. a, Protein from the flow-through (lane I) and eluted fractions (lane II) are individual western blots of the IgG subclasses. HRP conjugated anti-human IgG subclass specific antibodies were used. **b**, DNA hydrolysis by IgG subclass fractions. Flow-through (lane I) and eluted fraction (lane II). UC-scDNA incubated alone; sc-Supercoiled DNA; rc- Relaxed circular DNA and ln- Linear DNA.
