# Supplementary material for: DNA hydrolysing IgG catalytic antibodies: an emerging link between psychoses and autoimmunity
Source: NPJ Schizophr. 2021 Feb 26;7:13. doi: 10.1038/s41537-021-00143-6 (PMC7910540; doi:10.1038/s41537-021-00143-6)
Supplement: Supplementary file 2 — REPORTING SUMMARY [file 41537_2021_143_MOESM2_ESM.pdf]

nature research

Corresponding author(s): DBPR/NPJSCHZ00553

Last updated by author(s): Jan 7, 2021

Reporting Summary

Nature Research wishes to improve the reproducibility of the work that we publish. This form provides structure for consistency and transparency in reporting. For further information on Nature Research policies, see our Editorial Policies and the Editorial Policy Checklist.

Statistics

For all statistical analyses, confirm that the following items are present in the figure legend, table legend, main text, or Methods section.

n/a	Confirmed	
☐	☒	The exact sample size (n) for each experimental group/condition, given as a discrete number and unit of measurement
☐	☒	A statement on whether measurements were taken from distinct samples or whether the same sample was measured repeatedly
☐	☒	The statistical test(s) used AND whether they are one- or two-sided<br>*Only common tests should be described solely by name; describe more complex techniques in the Methods section.*
☒	☐	A description of all covariates tested
☐	☒	A description of any assumptions or corrections, such as tests of normality and adjustment for multiple comparisons
☐	☒	A full description of the statistical parameters including central tendency (e.g. means) or other basic estimates (e.g. regression coefficient) AND variation (e.g. standard deviation) or associated estimates of uncertainty (e.g. confidence intervals)
☐	☒	For null hypothesis testing, the test statistic (e.g. F, t, r) with confidence intervals, effect sizes, degrees of freedom and P value noted<br>*Give P values as exact values whenever suitable.*
☒	☐	For Bayesian analysis, information on the choice of priors and Markov chain Monte Carlo settings
☒	☐	For hierarchical and complex designs, identification of the appropriate level for tests and full reporting of outcomes
☒	☐	Estimates of effect sizes (e.g. Cohen's d, Pearson's r), indicating how they were calculated

Our web collection on statistics for biologists contains articles on many of the points above.

Software and code

Policy information about availability of computer code

Data collection	Microsoft Excel 2010
Data analysis	GraphPad Prism version 6

For manuscripts utilizing custom algorithms or software that are central to the research but not yet described in published literature, software must be made available to editors and reviewers. We strongly encourage code deposition in a community repository (e.g. GitHub). See the Nature Research guidelines for submitting code & software for further information.

Data

Policy information about availability of data

All manuscripts must include a data availability statement. This statement should provide the following information, where applicable:

- Accession codes, unique identifiers, or web links for publicly available datasets
- A list of figures that have associated raw data
- A description of any restrictions on data availability

The data used in this study is available with the corresponding author on a reasonable request and will be made available with a prior permission from the JIPMER, India.

Field-specific reporting

Please select the one below that is the best fit for your research. If you are not sure, read the appropriate sections before making your selection.

☒ Life sciences ☐ Behavioural & social sciences ☐ Ecological, evolutionary & environmental sciences

For a reference copy of the document with all sections, see nature.com/documents/nr-reporting-summary-flat.pdf

Life sciences study design

All studies must disclose on these points even when the disclosure is negative.

Sample size	No sample size was calculated. This was preliminary exploratory case-control study.
Data exclusions	No data was excluded.
Replication	The experiments were performed in duplicates.
Randomization	The samples were not randomized. This was an exploratory case-control study.
Blinding	The clinical data was blinded to the biochemist group until the DNA activity analyses was completed.

Reporting for specific materials, systems and methods

We require information from authors about some types of materials, experimental systems and methods used in many studies. Here, indicate whether each material, system or method listed is relevant to your study. If you are not sure if a list item applies to your research, read the appropriate section before selecting a response.

Materials & experimental systems

n/a	Involved in the study	
☐	☒	Antibodies
☒	☐	Eukaryotic cell lines
☒	☐	Palaeontology and archaeology
☒	☐	Animals and other organisms
☐	☒	Human research participants
☒	☐	Clinical data
☒	☐	Dual use research of concern

Methods

n/a	Involved in the study	
☒	☐	ChIP-seq
☒	☐	Flow cytometry
☒	☐	MRI-based neuroimaging

Antibodies

Antibodies used	SIGMA-ALDRICH, USA<br>Rabbit Anti-Human IgG (whole molecule)–Peroxidase antibody , Product no: A8792<br><br>P.A.R.I.S, France<br>Goat Anti-Human IgG (gamma chain specific) peroxidase conjugate, Product no: BI2418<br><br>JANSSEN BIOCHIMICA, Belgium<br>Mouse Monoclonal Anti-human IgG1 HRP conjugate, Product no: 2414589<br>Mouse Monoclonal Anti-human IgG2 HRP conjugate, Product no: 2414488<br>Mouse Monoclonal Anti-human IgG3 HRP conjugate, Product no: 2414387<br>Mouse Monoclonal Anti-human IgG4 HRP conjugate, Product no: 2414286
Validation	N/A and only secondary Abs were used.

Human research participants

Policy information about studies involving human research participants

Population characteristics	Patients diagnosed as SCZ (n=31) and BPD (n=31), respectively and who fulfilled the study inclusion criteria - Diagnostics and Statistical Manual of Mental disorder, IV edition (DSM IV) criteria, absence of any autoimmune disorder, inflammatory and neurological disorders were only recruited.<br>A subset of Systemic lupus patients diagnosed with neuropsychiatric symptoms (n=20) and healthy controls (n=25) were also enrolled. All the samples were of south Indian ethnicity

Recruitment	The participants for the study were selected from individuals attending out-patient or in-patient services at the Department of Psychiatry, at JIPMER, India. The neuropsychiatric symptoms systemic lupus patients were recruited at Department of Clinical Immunology at JIPMER, India
Ethics oversight	The study protocol was approved by Institutional Ethics committee of JIPMER, India (ECR/342/Inst/PY/2013) and Vellore Institute of Technology (VIT), Vellore (IECH/2014/May23/05). Written informed consent was obtained from all the participants.

Note that full information on the approval of the study protocol must also be provided in the manuscript.
